# Manipulation in Culture Conditions of *Nanofrustulum shiloi* for Enhanced Fucoxanthin Production and Isolation by Preparative Chromatography

**DOI:** 10.3390/molecules28041988

**Published:** 2023-02-20

**Authors:** Ayşegül Erdoğan, Ayça Büşra Karataş, Dilan Demir, Zeliha Demirel, Merve Aktürk, Öykü Çopur, Meltem Conk-Dalay

**Affiliations:** 1 Application and Research Centre for Testing and Analysis (EGE MATAL), Ege University, Bornova, 35100 İzmir, Turkey; 2 Department of Bioengineering, Faculty of Engineering, Ege University, Bornova, 35100 İzmir, Turkey; 3 Department of Chemistry, Faculty of Science, Ege University, Bornova, 35100 İzmir, Turkey

**Keywords:** fucoxanthin, diatom, extraction, purification, preparative chromatography

## Abstract

Microalgae produce a variety of high-value chemicals including carotenoids. Fucoxanthin is also a carotenoid that has many physiological functions and biological properties. For this reason, the cost-effective production of fucoxanthin at an industrial scale has gained significant attention. In the proposed study, fucoxanthin production was aimed to be increased by altering the culture conditions of *N. shiloi.* The effect of light intensity aeration rate, different nitrogen sources, and oxidative stress on the biomass and fucoxanthin productivity have been discussed. Based on these results, the fucoxanthin increased to 97.45 ± 2.64 mg/g by adjusting the light intensity to 50 µmol/m^2^s, and aeration rate at 5 L/min using oxidative stress through the addition of 0.1 mM H_2_O_2_ and 0.1 mM NaOCl to the culture medium. Fucoxanthin was then purified with preparative HPLC using C_30_ carotenoid column (10 mm × 250 mm, 5 μm). After the purification procedure, Liquid chromatography tandem mass spectrometry (LC–MS/MS) and UV-vis spectroscopy were employed for the confirmation of fucoxanthin. This study presented a protocol for obtaining and purifying considerable amounts of biomass and fucoxanthin from diatom by manipulating culture conditions. With the developed methodology, *N. shiloi* could be evaluated as a promising source of fucoxanthin at the industrial scale for food, feed, cosmetic, and pharmaceutical industries.

## 1. Introduction

There is growing interest in microalgae as a source of high-value compounds in the food and medicinal industries. The natural metabolites obtained from microalgae are the most important field of study in microalgal biotechnology. The pigments, vitamins, proteins, and fatty acids they contain are widely used in agriculture, medicine, pharmacy, and the cosmetics sector [1,2]. Particularly, the reasons why microalgae are preferred in this regard can be summarized as being able to increase their daily weight rapidly, easily undergo biotechnological processes, have low production costs, and be resistant to environmental effects [3]. Most of the products beneficial to humans emerge through biotechnological methods [4,5]. For this reason, researchers mainly focus on biotechnologically obtained products rather than synthetic materials for many reasons. Pigments are also important metabolites that have gained importance in recent years. Microalgae are seen as potential organisms in the production of pigments [6]. The formation of free radicals that cause cancer, and a number of chronic diseases that cause various damages to the organism as a result of oxidation during the functioning of the metabolism has increased interest in antioxidant compounds [7]. Carotenoids, which are among the photosynthetic pigments of microalgae, also have a strong antioxidant effect. Carotenoids are a class of oil pigments that can only be produced by phytoplankton algae, plants, and a limited number of fungi and bacteria [8]. They are fat-soluble pigments that give colour to various vegetables, fruits, beverages, flowers, mushrooms, algae, and even birds. Colours range from light yellow to bright orange and red depending on their chemical structure. In general, carotenoids in food are a category of tetraterpenoid (C_40_); it consists of eight isoprenoid units [9]. From the middle of the molecule, this structure has been reversed, and thus the molecule has gained a symmetrical structure [10]. Structures consisting of structural polyene chains can sometimes end in rings. Hydrocarbon-based carotenoids such as alpha-carotene, beta-carotene, and lycopene are called carotenes. On the other hand, lutein, zeaxanthin, and fucoxanthin belong to the xanthophylls group of carotenoids, as they also contain oxygen [11]. Fucoxanthin is a basic carotenoid found in brown algae, diatoms, and golden algae [12]. Besides, several researchers reported that fucoxanthin has no toxic effect and is a safe pharmaceutical active substance [13,14,15]. Due to its unique molecular structure, fucoxanthin has very important properties. It includes a polyene chain structure as well as functional groups such as epoxy, hydroxyl, carbonyl, and carboxyl. Many benefits of fucoxanthin have been reported for human health. The most important of these are their anti-cancer, anti-hypertensive, anti-inflammatory, and anti-obesity therapeutic properties [16,17,18]. For the industrial production of fucoxanthin, brown macroalgae are used in most studies [19,20,21]. Many studies have indicated that the content of fucoxanthin in microalgae is higher than in macroalgae. In chromist marine microalgae, the amount of fucoxanthin accounts for about 1–2.5% of the dry cell weight, while in brown macroalgae, the content of fucoxanthin is nearly 0.1–1 mg/g (dry cell weight). Brown macroalgae are presently sold as a commercial source of healthy pigment. Notwithstanding, macroalgal production is not an economically viable source due to the low extraction efficiency and slow growth, which is seasonally dependent [22,23]. Therefore, it does not seem appropriate for them to be commercially produced. The production of microalgae is easier and faster. This is because they can double their weight in a very short time. In fact, growth rates increase logarithmically. Today, microalgal biotechnology is progressing rapidly, but there is still a great demand for the commercial production of fucoxanthin from microalgae. Fucoxanthin production is dependent on microalgae type, growth, fucoxanthin concentration, and extraction efficiency. The microalgae’s fucoxanthin synthesis varied depending on the growing conditions, light intensity, temperature, nitrogen source and/or concentration iron and silicate concentration, carbon dioxide, salinity etc. [24] Some microalgal species have a rather high fucoxanthin content (without ideal growth conditions), ranging from 0.22–1.82% of the total dry weight (DW). Recently, discussion of topics such as culture conditions, heterotrophic and mixotrophic cultivation, the use of low-cost by-products as nutrient media, and pilot-scale studies in the cultivation of fucoxanthin accumulating algae has become very popular. In addition to nitrogen, iron, and silicate concentrations, light intensity, and wavelength have a very important effect on the biosynthesis of fucoxanthin, and the production of biomass in microalgae. With their quick growth rate and high fucoxanthin content, microalgae such as *Isochrysis* spp. (7.5–23.3 mg/g), *Nitzschia* spp. (12–32.8 mg/g), *Phaeodactylum tricornutum* (10.9–59.2 mg/g), *Tisochrysis lutea* (2.1–79.4 mg/g), and others are a promising source for fucoxanthin production [25,26,27,28,29,30,31,32]. With the current findings and recommendations, the economic viability of fucoxanthin production might be improved, possibly leading to a feasible and sustainable production method [33].

When literature studies are examined, most of them focus on the bioactivities of fucoxanthin [12,17,33,34,35,36,37,38]. Studies on microalgae production (for example, optimization of culture conditions, and attempts for pilot scale work) and the use of advanced extraction technologies to obtain fucoxanthin have rarely been discussed [24]. As this information is valuable for future commercial scale production of fucoxanthin, this study demonstrates that fucoxanthin-producing *N. shiloi* can provide significant increases in both biomass and fucoxanthin content when grown under different culture conditions. Studies on the use of low-cost by-products as a nutrient medium help to fill in gaps in the research in the production of fucoxanthin. In addition, discussions were made on the mechanisms underlying the production of fucoxanthin using different culture conditions in order to guide future studies. This study aimed to present the enhanced productivity of fucoxanthin from *N. shiloi* by altering the light intensity, aeration rate, nitrogen source, and applying oxidative stress to culture conditions. After that, fucoxanthin was purified by preparative chromatography. The purity of fucoxanthin was proven by UV-vis spectroscopic and mass spectrometric data. Here, it was shown that, by altering culture conditions, it was possible to increase the fucoxanthin content. *N. shiloi* was shown to be a novel diatom and could serve as a potential source for fucoxanthin production at an industrial level.

## 2. Results

### 2.1. Enhanced Productivity of Fucoxanthin

The growth profile and fucoxanthin concentration of *N. shiloi* were investigated to cultivate under different physical environmental conditions. It is well-known that light intensity, aeration rate, nitrogen sources, and oxidative stressors are factors that affect growth and pigment biosynthesis in microalgae. 

**Figure 1:**
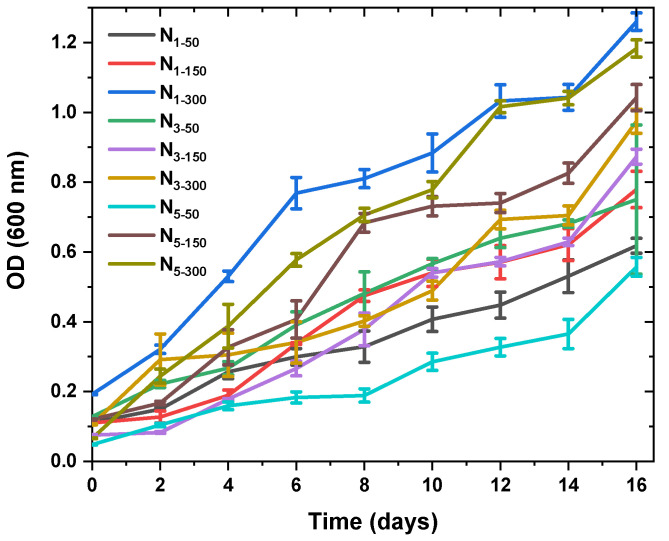
Plot showing the effect of light intensities and aeration rates on the optical density of *N. shiloi*. Reported results are the average of three replicates (i.e., n = 3), with error bars denoting one standard deviation about the mean.

Figure 1 shows that biomass productivity and doubling rates are greater at 300 µmol/m^2^ of light intensity compared to 50 and 150 µmol/m^2^. According to Table 1, the increase in biomass was the highest at 300 µmol/m^2^ light intensity. By increasing the aeration rate and light intensity, the mixing speed in the bioreactors was accelerated, and it was established that the suspension duration of the cell biomass increased the amount of biomass. The results emphasize that raising aeration rate and reducing the light intensity significantly increases the amount of fucoxanthin in the living cells.

The results showed that the three different light intensity and aeration rate factors had a significant effect on the fucoxanthin production of the diatom *N. shiloi.* The amount of fucoxanthin concentration obtained from *N. shiloi* enhanced with increasing light intensity at different aeration rates.

Table 1 illustrates that the highest amount of fucoxanthin (51.05 ± 1.02 mg/gDW) was obtained at a light intensity of 50 µmol/m^2^s and an aeration rate of 5 L/min in seawater BG11 medium (used NaNO_3_). For simplicity, aeration rates (1-3-5 L/min) and light intensities (50-150-300 µmol/m^2^s) were abbreviated, as given in Table 1. According to these results, low light intensity increases fucoxanthin concentration in *N. shiloi.*

**Figure 2:**
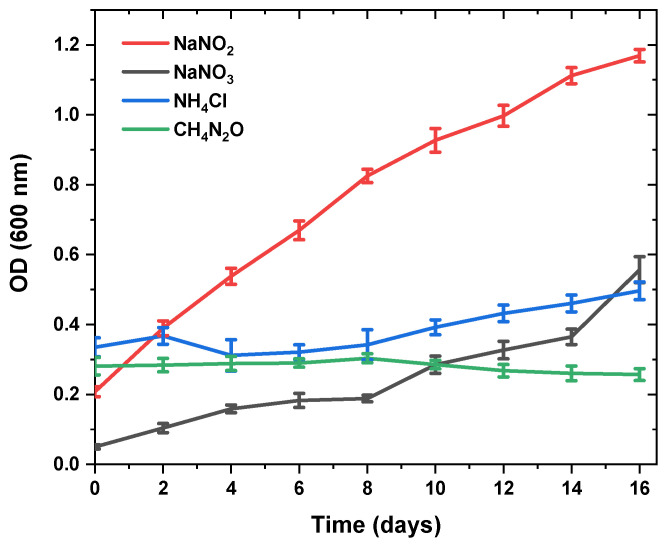
Plot showing the effect of different nitrogen sources on the optical density of *N. shiloi*. Reported results are the average of three replicates (i.e., n = 3), with error bars denoting one standard deviation about the mean.

On the other hand, fucoxanthin production significantly decreased when different nitrogen sources (NaNO_2_, NH_4_Cl and CH_4_N_2_O instead of NaNO_3_) were used in BG11 culture medium, separately. The growth curves of NaNO_3_, NaNO_2_, NH_4_Cl, and CH_4_N_2_O were monitored to determine the specific growth rates. Adding NH_4_Cl to a culture medium prevented *N. shiloi* from reproducing and growing. In contrast to the control (NaNO_3_), the culture to which CH_4_N_2_O was introduced was not a brown color, and phase-contrast microscopy revealed the presence of empty silica walls. Particularly, the use of urea caused the cell death of *N. shiloi*. It took the cells a long time to adjust to the NH_4_Cl medium, and they were unable to finish their growth within 16 days. In addition, Figure 2 depicts the inability of cells in NaNO_2_ media to complete the logarithmic phase. 

The biomass productivity results and fucoxanthin yield are summarized in Table 2, where different nitrogen sources were used for the cultivation of *N. shiloi* under 50 μE/m^2^s light intensity and at a 5 L/min aeration rate. Based on these results, fucoxanthin could not accumulate excessively in the cells even though the NaNO_2_ medium was used.

The pH of the culture medium without *N. shiloi* (Day 1) and culture medium before harvesting the cells (Day 16) are measured and summarized in Table 3. It was seen that the pH dropped dramatically when NH_4_Cl and CH_4_N_2_O were used as nitrogen sources. 

**Figure 3:**
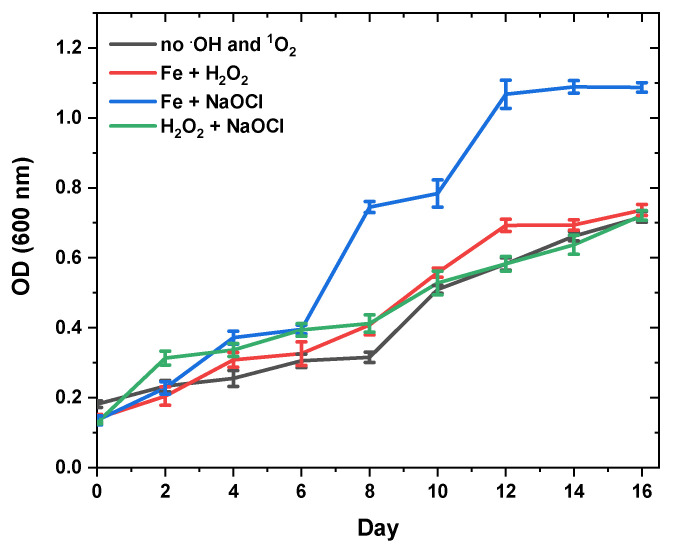
Plot showing the effect of various oxidative stress conditions on the optical density of *N. shiloi*. Reported results are the average of three replicates (i.e., n = 3), with error bars denoting one standard deviation about the mean.

The following step was the application of oxidative stress using various sources. Growth of *N. shiloi* was consistent for each case (Figure 3). Since the *N. shiloi* cultures were not axenic, the addition of Fe+NaOCl may have destroyed the bacteria in the environment. In addition, the addition of H_2_O_2_+NaOCl did not inhibit cell proliferation in comparison to the control group.

Finally, the effect of oxidative stress was investigated using H_2_O_2_, NaClO, and Fe^2+^. It was observed that fucoxanthin productivity reached the maximum (97.45 ± 2.64 mg/gDW) if 0.1 mM H_2_O_2_ was used with 0.1 mM NaOCl, while biomass productivity did not change significantly, as presented in Table 4. It was also apparent that each oxidative stress condition leads to the increase in the amount of fucoxanthin.

### 2.2. Morphological Changes in N. shiloi 

**Figure 4:**
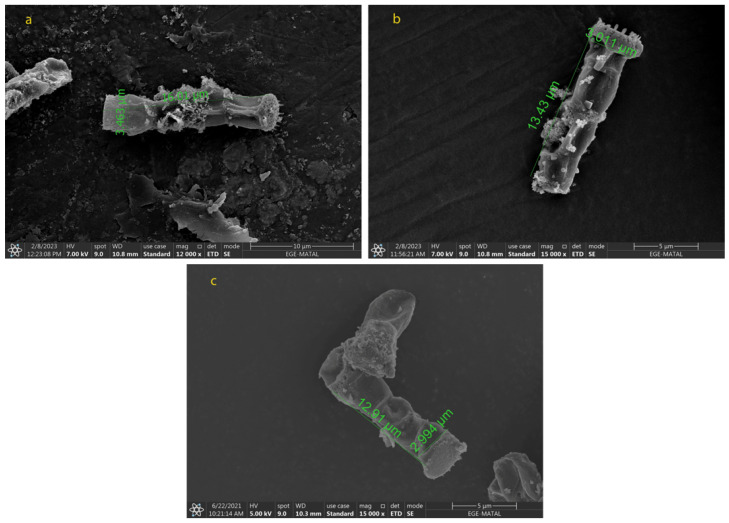
Morphological changes of *N. shiloi* in response to different light intensities (**a**) N_5-50_ (**b**) N_5-300_ and (**c**) N_5-150_.

**Figure 5:**
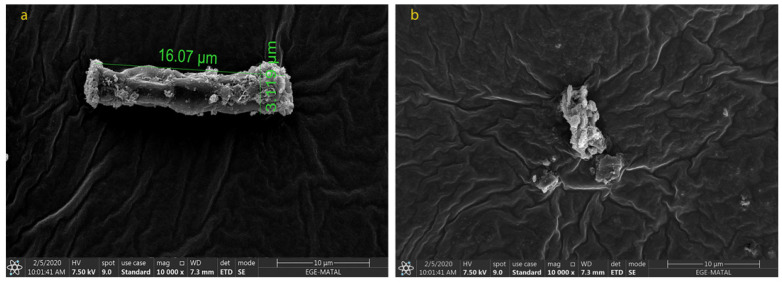
Morphological changes of *N. shiloi* in response to different nitrogen sources (**a**) NaNO_3_ and (**b**) CH_4_N_2_O.

**Figure 6:**
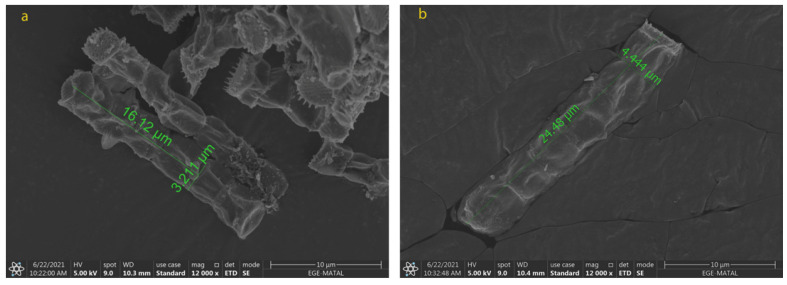
Morphological changes of *N. shiloi* in response to oxidative stress (**a**) no ^.^OH and ^1^O_2_ (**b**) 0.1 mM H_2_O_2_ + 0.1 mM NaOCl.

The cell size and shape directly affect the extraction of biomolecules from each species. To obtain the carotenoids efficiently after the extraction processes, the cell wall of the microalga should be destroyed and dissolved in the appropriate solvent. It is particularly important in repetitive extractions, as the number of extractions increases the cost as well. Due to the structure of *N. shiloi*, fucoxanthin could be easily extracted by the UAE method. It was reported that *Navicula* sp. cell size showed a change under indoor and outdoor cultivation conditions [39]. The fucoxanthin of *Navicula* sp. reduced between 5.40 ± 0.05 (indoor) and 2.61 ± 0.06 mg/gDW (outdoor) of biomass. The cultivation of *Amphora* sp. was incubated in the optimum conditions at 30 °C and 80 μmol/m^2^s [40]. In the present study, it was observed from the SEM results that the cell size of *N. shiloi* also changed with different culture conditions. The variation in light intensity and aeration rate triggered the morphological changes in *N. shiloi*. The maximum cell size was observed for N_5-50_ (16.17 ± 0.25 µm × 3.476 ± 0.028 µm). The size of the cells decreased when higher light intensities were applied (Figure 4). On the other hand, the use of different nitrogen sources negatively affected the productivity and morphology of *N. shiloi.* The cells cultured in urea were dead, and no uniform cells were observed (Figure 5). Particularly, the diatom filament wall size was measured as 12.75 ± 0.29 µm × 2.955 ± 0.057 µm without any oxidative stress, and 24.86 ± 0.69 µm × 4.273 ± 0.303 µm in the presence of 0.1 mM H_2_O_2_, and 0.1 mM NaOCl in culture medium, as presented in Figure 6. This corresponds to approximately a 2-fold increase in both the width and height of *N. shiloi* cells.

### 2.3. Fucoxanthin Analysis and Isolation by Preparative Chromatography

**Figure 7:**
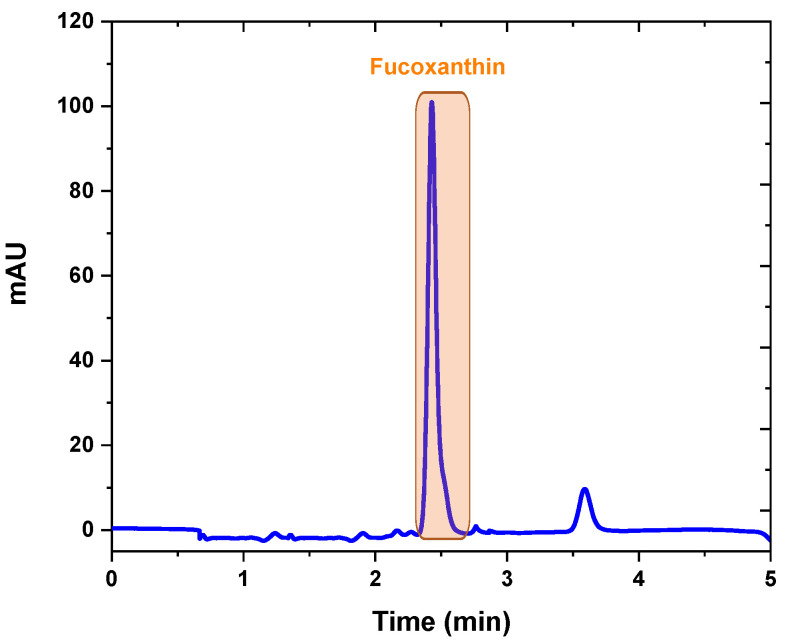
Prep-HPLC chromatogram for *N. shiloi* extract obtained at 450 nm.

Quantification of fucoxanthin was realized by HPLC–DAD with a simple and fast isocratic elution with a modified protocol proposed by Erdoğan, et al. [41] using Methanol: Acetonitrile (85:15) with a flow rate of 1.2 mL/min. Fucoxanthin was isolated by preparative chromatography rapidly with a higher flow rate (4 mL/min) so that fucoxanthin can be collected with sequential injections in a shorter time. No serious problems were encountered in the separation of fucoxanthin by prep-HPLC. The fucoxanthin peak was collected between 2.22 and 2.73 min, as presented in Figure 7. Each fraction was placed in an amber bottle and flushed with nitrogen gas to avoid degradation of fucoxanthin due to light and oxygen.

### 2.4. Confirmation of Purified Fucoxanthin with UV-vis Spectroscopy and Mass Spectrometry 

**Figure 8:**
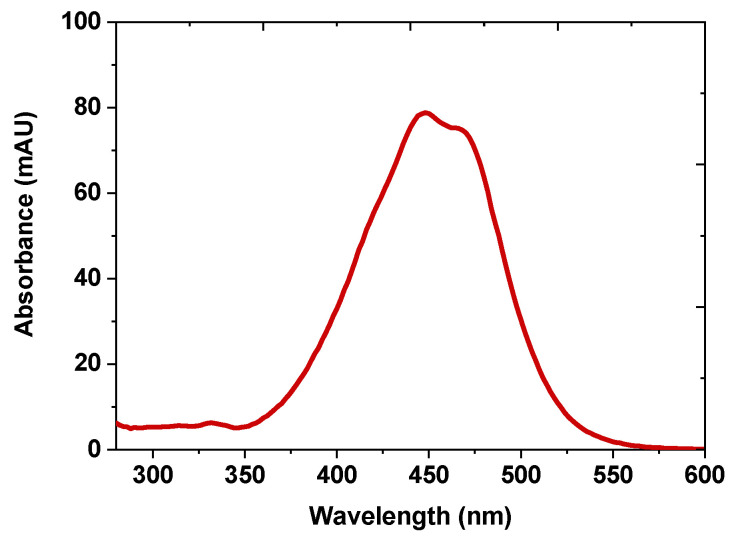
UV-vis spectra of purified fucoxanthin form *N. shiloi*.

Purified fucoxanthin was dissolved in mobile phase and injected to HPLC-DAD. The absorbance values between 300 and 600 nm were collected during HPLC-DAD analysis. According to the data obtained, fucoxanthin fine structure and absorbance values are in agreement with the literature values, as depicted in Figure 8 [42].

**Figure 9:**
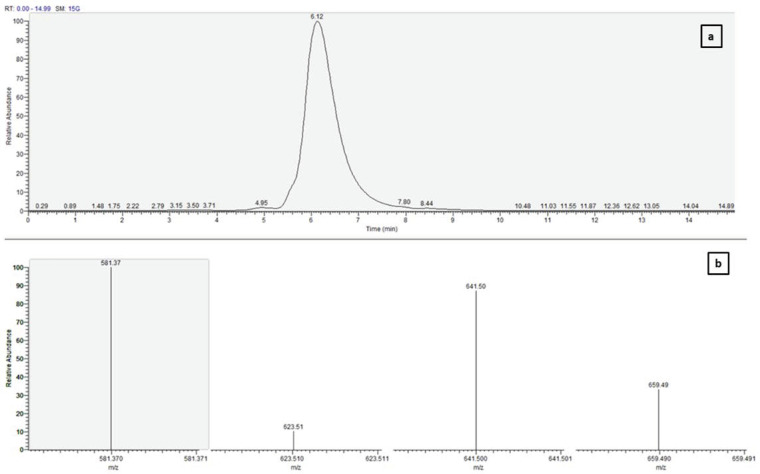
Liquid chromatography mass spectrometric data for fucoxanthin (**a**) LC chromatogram and (**b**) LC–MS/MS spectra for fucoxanthin purified from *N. shiloi* after preparative chromatography.

The collected fraction after preparative chromatography was dissolved in 10.0 mL of methanol and subjected to LC–MS/MS analysis for molecular weight. Fucoxanthin was also identified and confirmed by retention time (RT) and Selected Ion Monitoring (SIM) mode. Analysis was performed in positive ion mode and optimized using commercial fucoxanthin standard. Figure 9 demonstrates the LC-MS/MS data for purified fucoxanthin from *N. shiloi.* Monitoring the ions m/z 659.49 [M+H]^+^, 641.50 [M+H-18]^+^, 622.51 [M+H-18-18]^+^, and 581.37 [M+H-18-60]^+^ confirms that fucoxanthin was separated, which is consistent with the literature values [42]. 

## 3. Discussion

### 3.1. Variation of Fucoxanthin Content and Biomass Productivity under Different Light Intensities and Aeration Dates

The growth rate of *N. shiloi* in F/2 medium, and the fucoxanthin content varied significantly under different light intensities and aeration rates. Three types of light responses as low light (50 μmol/m^2^s), medium light (150 μmol/m^2^s), and high light (300 μmol/m^2^s) with varying aeration rates (1-3-5 L/min.) were investigated since the light is one of the most important parameters that influence the amount of carotenoids produced in microalgae. In *N. shiloi*, low intensities of light caused an increase in the amount of fucoxanthin. Although the adaptability of microalgae to environmental stress, particularly light intensity, is species-specific, there is a similar trend among fucoxanthin-producing microalgae, which states that low light intensity induces a higher accumulation of fucoxanthin [32]. This increase may be due to the Fucoxanthin–chlorophyll proteins (FCP) which are associated with photosystems (PS) I and II in the thylakoid membranes (TM) in algae. The FCP complex is responsible for light-harvesting and electron transfer reactions during photosynthesis [43,44,45]. Apart from light harvesting capacity, FCPs have been also investigated for the photoprotective capacity [46,47]. Under low light (LL) conditions, diatoms require more FCP complexes to compensate for the reduction in photon flux [45]. Thus, photosynthetic pigments including FX and FCP antennae should be provided to form more FCP complexes. In this case, more FCP complexes or larger complexes may be required to overcome the lack of photon flux under LL. In contrast to LL condition, excess photon flux must switch on the photoprotective mechanism to prevent photoinhibition and photodamage, which can threaten microalgal viability [48]. High light (HL) stress may cause alterations in the photosynthetic apparatus, such as chloroplast fragmentation and TM degradation, leading to an increase in secondary carotenoids (DD and DT) rather than in the primary carotenoid FX [49].

When different aeration rates and light intensities are applied, the best result is found in N_5-50_. Although the amount of biomass is not as high compared to other conditions, the content of fucoxanthin is the highest as 51.05 ± 1.02 mg/gDW. Additionally, it is very important to reduce the steps during purification since the purification step will be required to commercially obtain fucoxanthin. For example, the amount of fucoxanthin obtained in N_5-50_ is 34% higher than in N_3-50_, and the growth rate is almost the same (Table 1). 

As previously reported in the literature, algal fucoxanthin production was found to be higher under low light intensities [50,51,52,53]. It has been hypothesized that the fucoxanthin variation caused by light may be related to the modulation of Diadinoxanthin Cycle [53,54,55]. When the studies in the literature are examined, it has been reported that the light intensity required for the production of high amounts of fucoxanthin varies between 10 and 100 μmol/m^2^s. Similarly, *N. shiloi* produced the maximum amount of fucoxanthin at 50 μmol/m^2^s light intensity in this study. Therefore, the obtained result is as expected [32].

In order to cope with the abiotic stress caused by the high light intensities, diatom cells tend to convert diadinoxanthin to diatoxanthin at the expense of fucoxanthin, leading to reduced fucoxanthin biosynthesis. However, it should be noted that this is dependent on the species and/or strains. Each microalga has its own capability to overcome the stress conditions or adaptation to different culture conditions [54].

### 3.2. Changes in Fucoxanthin Content and Biomass Productivity Using Different Nitrogen Sources 

The effects of various nitrogen sources on the development and generation of fucoxanthin in microalgae were also studied [56,57,58]. For this reason, *N. shiloi* cells were cultivated in seawater BG11 medium in which the nitrogen sources were varied. Apart from nitrate, sodium nitrite, ammonium chloride, and urea were used at the same concentrations. The maximum specific growth rate was highest and the same when nitrate and nitrite were used as nitrogen sources. However, cell death was observed when ammonium and urea were used instead. These results have indicated that *N. shiloi* could not compensate the pH changes. Nitrogen is an important element for growth, and while microalgae can utilize a range of nitrogenous compounds, it has been shown that ammonium ions, if present above a threshold level, are assimilated in preference to other nitrogen sources [59,60,61,62]. The assimilation of various nitrogen sources can have a pronounced effect on the pH of the medium in non-pH-stat cultures.

The extent and direction of the pH change is likely to be due to the rate and form of the assimilated nitrogen, and the buffering capacity of the medium. Apart from the direct energy requirements for assimilating the different nitrogen sources, the observed pH changes may have, in turn, altered the maximum specific growth rate and fucoxanthin. 

It is unknown if the higher concentrations of ammonium chloride and urea inhibited the cell division or if they were toxic to the cells. Toxic effects of ammonium ions and urea are often attributed to ammonia (NH_3_), which may accumulate from the decomposition of urea or from the dissociation of ammonium ions. The potential toxicity could be clarified if, in the future, the concentration of ammonia in the cultivation media is monitored and cell viability assays are undertaken. The increase in the maximum specific growth rate of *C. cryptica* when cultivated with low concentrations of ammonium chloride or urea could be a direct result of the assimilation efficiency of ammonium ions or urea (as less energy is required for the assimilation of ammonium and urea), or an indirect result based on the pH of the cultivation media. While the preferential assimilation of ammonium ions or urea does not necessarily imply faster growth (growth rate being restricted by the rate-limiting step within the system), the source of nitrogen may alter the way metabolic energy is spent. However, the addition of different nitrogen sources should be more explored and optimized due to the potential toxic effects of ammonium and ammonia concentrations derived from urea conversion [63]. Our experiments show that the addition of different nitrogen sources decreased the fucoxanthin productivity and cell density as compared with cultivation with nitrate. 

Possibly, high urea concentrations might lead to growth reduction through toxicity effects. Previous researchers demonstrated that microalgae could assimilate urea as a source of nitrogen by converting it to NH_4_^+^ and CO_2_ through urease activity, or ammonium and bicarbonate via ATP-urea amidolyase [64,65]. This might lead to high ammonium or ammonia concentrations, which tend to be toxic for diatoms such as *P. tricornutum*, as well as other algae such as chlorophytes [66,67]. Consequently, commercial nitrates such as NaNO_3_ are still recommended for microalgal culture, despite the considerable need for a substitute nitrogen source that is less expensive.

### 3.3. Effect of Oxidative Stress on Fucoxanthin Content and Biomass Productivity

Reactive oxygen species (ROS) are continuously produced in microalgae, chloroplasts, mitochondria, and peroxisomes. ROS production and cleaning must be balanced so that it does not damage cell components. Therefore, antioxidant protective mechanisms are generally engaged [68]. Hydrogen peroxide, a type of ROS, is also a microalgae product released through oxidative metabolism. Almost all living things decompose hydrogen peroxide into water and oxygen at low concentrations. H_2_O_2_ can damage cells at high concentrations, but moderate levels of cells can adapt to this condition. Hydrogen peroxide decomposes, especially in the presence of iron, and forms the highly reactive hydroxyl radical through the Fenton reaction. Uncontrolled production of ROS can also destroy proteins, lipids, and carotenoids. Therefore, as in many other organisms, microalgae develop defence mechanisms to deal with this situation when faced with high ROS levels [69]. One of them is that, in the presence of ROS, it increases the production of carotenoids with high antioxidant activity in order to protect the cells against oxidative damage.

Unfortunately, there is not enough information in the literature about the stimulating mechanism of fucoxanthin formation by ROS. In some of the studies, the production of fucoxanthin was triggered in *C. closterium* and *A. capitellata* again by using the same types of ROS [41,70]. 

In this study, an increase in fucoxanthin accumulation was also observed by using ROS concentrations at the same concentrations. In Table 2, it can be said that the accumulation of fucoxanthin in *N. shiloi* is remarkable. In the presence of 0.1 mM H_2_O_2_ + 0.1 mM Fe^2+^, the amount of fucoxanthin increased slightly (58.20 ± 1.16 mg/gDW) compared to the control group (50.17 ± 1.02 mg/gDW). This value increased to 65.14 ± 1.95 mg/gDW in the presence of 0.1 mM NaClO + 0.1 mM Fe^2+^. When 0.1 mM H_2_O_2_ + 0.1 mM NaOCl was used, the fucoxanthin value almost doubled (97.45 ± 2.64 mg/gDW). When the data are examined, it is thought that H_2_O_2_ and NaClO are very effective on fucoxanthin accumulation and the presence of Fe^2+^ does not contribute much. When H_2_O_2_ was used in combination with NaClO, the remarkable increase in the amount of fucoxanthin supports this. Probably *N. shiloi* increased the accumulation of fucoxanthin to cope with this stress at the given concentrations.

When the stress sources were examined, it was seen that there was only a partial decrease in the specific growth rate of *N. shiloi* in only the first two stress sources. In other words, it can be said that these two sources for the concentrations used create stress for *N. shiloi.* However, the most notable of the results here is that, when H_2_O_2_ and NaClO are used together, there is no reduction in growth rate and even remain the same. This shows that microalgae do not accept H_2_O_2_ and NaClO as stress factors at given concentrations, and at least produces high amounts of fucoxanthin and easily copes with this stress.

It seems that this species has easily adapted itself to oxidative stress conditions by activating defence mechanisms [71]. As with all living things, microalgae have several defence systems, both enzymatic and non-enzymatic, for detoxification of ROS. In the literature, it has been reported that three reaction mechanisms describe the reaction of free radicals with carotenoids, namely, electron transfer, hydrogen atom transfer, and radical addition to carotenoids [72,73]. To remove free radicals, carotenoids can donate or accept unpaired electrons. Generally, antioxidant molecules are oxidized by giving electrons to free radicals. However, carotenoids can absorb free radicals by accepting an unpaired electron, making it harmless by translocation on the conjugated side chain. For these reasons, it may be possible to induce the synthesis of fucoxanthin during quenching with ROS.

## 4. Materials and Methods

### 4.1. Reagents and Chemicals

All-trans-fucoxanthin, triethylamine, pyrogallol, silica gel, and calcium carbonate were provided by Sigma-Aldrich, and all the solvents used in this study were LC-grade purchased from Merck.

### 4.2. Molecular Identification of N. shiloi

The benthic diatom *N. shiloi* was previously identified, and the details are given by Demirel, et al. [74]. In that study, benthic diatoms were separated from the Aegean Sea in Türkiye and identified according to their morphological characteristics on the basis of observations in bright field and scanning electron microscopy. For DNA extraction, the cultured strain was harvested by centrifugation, and the cell pellet was used in the DNA Kits (Zymo Research) and stored at −20 °C. PCR primers targeted to link 18S rDNA (F, 5′-YACCTGGTTGATCCTGCCAGTAG-3′ and R, 5′-GCTTGATCCTTCTGCAGGTTCACC-3′). PCR protocol was applied, and products were viewed in an agarose gel stained by Jel Safe Dye and checked the gel under a UV light. In the DNA sequencing step, dye-terminator sequencing was performed using the primers, and the nucleotide chromatograms were determined by DNA sequences. The sequences obtained in the mentioned study were deposited in the NCBI GenBank, and the accession numbers of KR149459. *Nanofrustulum shiloi* (EGEMACC 47) was deposited in Ege University, Microalgae Culture Collection, Izmir, Turkey (EGEMACC—https://ege-macc.ege.edu.tr/ (accessed on 15 February 2023)).

### 4.3. Cultivation and Harvesting of N. shiloi

*N. shiloi* was cultivated in BG11 medium based on artificial seawater [73] at 20 ± 2 °C under continued light intensity. The diatom was added in a bubble-column photobioreactor (BCP, 2 L) and cultivated under the light intensity of 50, 150, and 300 µmol/m^2^s (Lutron LX-1108, Taiwan), at 21 ± 2 °C, and with the aeration rate of 1, 3, and 5 L/min. The growth of microalgae was pursued by counting microalgae cells optical density at 2-day intervals over the period of 16 days. The specific growth rate for *N. shiloi* was calculated according to Becker [75] (Equation (1)) using the data obtained by the absorbance values taken at 600 nm.

(1) $$\mu \;=\frac{\mathrm{lnx}2-\ln x1}{\Delta t}$$

where μ = specific growth rate, x_2_ = cell concentration at time, t_2_, x_1_ = cell concentration at time t_1_, and Δt = t_2_ − t_1_. Doubling time was determined as 0.693/µ. 

The recipe of seawater BG11 was prepared in 1.5 g/L sodium nitrate (NaNO_3_-17.65 mM), sodium nitrite (NaNO_2_), ammonium chloride (NH_4_Cl), and urea (CH_4_N_2_O), and were calculated at the same concentrations, separately. 

In the control group, the culture medium was prepared using 1.5 g/L sodium nitrate (NaNO_3_-17.65 mM), as recommended in the Seawater BG11 recipe (American Type Culture Collection, https://www.atcc.org/search#q=bg%2011&sort=relevancy&numberOfResults=24&f:Documenttype=[Media%20formulation] (accessed on 15 February 2023). In order to examine the effect of different nitrogen sources, it was prepared separately with sodium nitrite (NaNO_2_), ammonium chloride (NH_4_Cl), and urea (CH_4_N_2_O) instead of sodium nitrate, with a constant nitrogen concentration of 17.65 mM.

*N. shiloi* was grown in a BCP, prepared with different nitrogen sources medium, and then cultivated under the light intensity of 50 µmol/m^2^s, at 21 ± 2 °C, and with the aeration rate of 5 L/min. 

Oxidative stress was created by reactive oxygen species via a series of chemical reactions, as Strati and Oreopoulou 2011 and de Almeida Torres et al. 2018 proposed. The different chemicals (H_2_O_2_, NaOCl and FeSO_4_) of oxidative stress were added to the culture media at a concentration of 0.1 mM. H_2_O_2_, NaOCl, and FeSO_4_, and were added to the culture media at constant concentrations of 0.1 mM in order to enable the release of reactive oxygen species through the 3 reaction templates given in R1, R2 and R3 [76,77].

R1: Fe^2+^ + H_2_O_2_ → Fe^3+^ + OH^−^ + ∙OH
R2: NaClO + H_2_O → HOCl + Na^+^ + OH^−^ and HOCl + Fe^2+^ → Fe^3+^ + Cl^−^ + ∙OH
R3: H_2_O_2_ + NaClO → H_2_O + NaCl + ^1^O_2_

The control group was cultured in seawater BG11 medium and then cultivated under the light intensity of 50 µmol/m^2^s, at 21 ± 2 °C, with the aeration rate of 5 L/min, and in BCP. Microalgal cells reached the stationary phase harvested by centrifugation (Pro Research, By Centurion Scientific Ltd., Chichester, UK). After, the pellet was washed with deionized water to remove the growing medium. Then, the paste biomass was lyophilized (Christ Alpha 1-2 LD plus, Osterode am Harz, Germany) and grounded using a mortar for the extraction process. They were stored at −20 °C under dark conditions. The changeable morphology of *N. shiloi* was examined by scanning electron microscopy (SEM) where characterizations were carried out by using Thermo Scientific Apreo S (Thermo Fisher Scientific, Waltham, MA, USA).

### 4.4. Preparation of Stock and Standard Fucoxanthin Solutions

For the preparation of 100.0 mg/L stock fucoxanthin solution, 1.0 mg of fucoxanthin was weighed and dissolved in 10.0 mL chloroform (stabilized with 1% ethanol). Calibration standards were prepared by using this stock solution for the construction of a calibration curve. All standard solutions were kept in amber-colored volumetric flasks. Different concentrations (0.05–10.0 mg/L) of fucoxanthin standards were injected into the HPLC-DAD.

### 4.5. Ultrasound-Assisted Extraction (UAE) of Fucoxanthin from N. shiloi

The extraction of fucoxanthin was performed by Ultrasound-Assisted Extraction (UAE) method by using an ultrasonic bath (Elmasonic, S80H). Ethanol was preferred as an extraction solvent, as it is environmentally preferable and referred to as “green”. Saponification procedure was eliminated, as the saponification causes fucoxanthin to turn into fucoxanthinol under basic conditions [44].

For the extraction process, the biomass (0.20 g) was added with CaCO_3_ (0.20 g) and 10.0 mL of ethanol containing 0.01% (*w*/*v*) pyrogallol. Then, the extraction was conducted by an ultrasonic bath for 15 min at 40 °C. After the UAE process, the solution was centrifuged at 5000 rpm for 2 min and the supernatant was filtered by 47 mm of 0.20 µm nylon filter paper (Sartorius, Goettingen, Germany). Then, the solution was diluted with the mobile phase (%70 Methanol and 30% Acetonitrile containing 0.01% *v*/*v* triethylamine) prior to HPLC-DAD analysis [43]. 

### 4.6. HPLC-DAD and LC-APCI-MS/MS Analyses of Fucoxanthin 

Fucoxanthin was quantified by HPLC 1260 Series (Agilent, Santa Clara, CA, USA) equipped with diode array detector at 450 nm with a flow rate of 1.2 mL/min using Waters YMC C_30_ Carotenoid column (4.6 mm × 250 mm, 5 µm). The mobile phase consisted of 70% methanol and 30% acetonitrile each including 0.01% triethyl amine (TEA) as a modifier. Fucoxanthin was purified by preparative HPLC (Thermo Scientific/Dionex Ultimate 3000). The preparative purification was performed with a YMC-C_30_ semi-prep carotenoid column (10 mm × 250 mm, 5 μm) using the same mobile phase composition (85:15, methanol: acetonitrile) at a flow rate of 4.0 mL/min at 450 nm with a column temperature of 25 °C. The injection volume was 1.0 mL.

In order to obtain the absorption profile of fucoxanthin, spectroscopic data were recorded between 300 and 600 nm. After the purification procedure, the absorbance spectrum of fucoxanthin standard and purified fucoxanthin were compared (Data not shown). In the present study, fucoxanthin obtained from *N. shiloi* extract and the purified fucoxanthin were identified and confirmed by liquid chromatography-tandem mass spectrometry (LC-MS/MS) equipped with an Atmospheric Pressure Chemical Ionization probe (APCI). The mass spectrometer was operated in full scan mode from m/z 50-900 at 350 °C for vaporization temperature. 

### 4.7. Statistical Analysis

Each experiment was performed in triplicate. Tukey’s test at a reliability level (of *p* < 0.05) was utilized to find differences between treatment levels. Minitab software was utilized to conduct statistical analyses (V18, Minitab Inc., State College, PA, USA).

## 5. Conclusions

Microalgae have significant potential to produce value-added products such as lipids, proteins, carbohydrates, and carotenoids that play a vital role in human health. The use of carotenoids in the pharmaceutical, nutraceutical, and food industries makes them valuable components of microalgae. As microalgae exhibit adaptation to different environmental factors such as temperature, light, salinity, etc., the production of carotenoids could be increased by changing the cultivation conditions. The results of this study showed that fucoxanthin content could be enhanced by altering the cultivation conditions. Moreover, it was proven that particularly the oxidative stress triggers the production of fucoxanthin in *N. shiloi* as in other diatoms. 

This article has discussed how to obtain high yields of fucoxanthin from *N. shiloi* as well as methodologies that can be used for new species. Combined with the new and innovative “green” solvent, ultrasound-assisted liquid extraction serves as an economically desirable and environmentally friendly extraction strategy. In addition, it is a pioneering work on obtaining fucoxanthin in a short time using the prep-column obtained with the latest technology. In the future, more intensive research, including pilot studies, should focus on photobioreactor-based bioengineering techniques, the integration of synergistic extraction strategies, and economic and environmental considerations, which are also suitable for industrial production for various purposes. 

## Figures and Tables

**Table 1:** Data for the production of *N. shiloi* and fucoxanthin amount under different light intensities and aeration rates.

* Aeration Rate and Light Intensity	Fucoxanthin (mg/gDW)	Specific Growth Rate (day^−1^)	Dry Weight (g/L)
N_1-50_	33.52 ± 0.67 ^b^	0.175 ± 0.03 ^a,b^	0.44 ± 0.02 ^e^
N_1-150_	10.86 ± 0.21 ^d,e^	0.203 ± 0.02 ^a,b^	0.24 ± 0.08 ^f^
N_1-300_	21.85 ± 0.43 ^e,f^	0.269 ± 0.02 ^a,b^	1.36 ± 0.08 ^a^
N_3-50_	38.06 ± 0.76 ^b^	0.160 ± 0.03 ^b^	0.52 ± 0.03 ^e^
N_3-150_	19.75 ± 0.39 ^f^	0.224 ± 0.03 ^a,b^	0.85 ± 0.07 ^d^
N_3-300_	26.79 ± 0.53 ^c,d^	0.188 ± 0.02 ^a,b^	1.05 ± 0.06 ^b,c^
N_5-50_	51.05 ± 1.02 ^a^	0.166 ± 0.01 ^b^	0.43 ± 0.09 ^e^
N_5-150_	23.47 ± 0.46 ^d,e^	0.330 ± 0.04 ^a^	0.95 ± 0.03 ^c,d^
N_5-300_	28.04 ± 0.56 ^c^	0.221 ± 0.02 ^b^	1.22 ± 0.08 ^a,b^

* Aeration rates (1-3-5 L/min) and Light intensities (50-150-300 µmol/m^2^s), ^abcdef^ Values for each stage within the same column bearing different superscripts are significantly different (*p* < 0.05).

**Table 2:** Data for the production of *N. shiloi* and variation in the fucoxanthin amount using different N-sources.

Nitrogen Sources	Fucoxanthin (mg/gDW)	Specific Growth Rate (day^−1^)	Dry Weight (g/L)
NaNO_3_	51.05 ± 1.58 ^a^	0.166 ± 0.04 ^a^	0.43 ± 0.09 ^b^
NaNO_2_	19.18± 0.39 ^b^	0.162 ± 0.03 ^a^	0.69 ± 0.07 ^a^
NH_4_ Cl	2.56 ± 0.04 ^c^	0.040 ± 0.00 ^b^	0.64 ± 0.08 ^a^
CH_4_N_2_O	-	-	-

^abc^ Values for each stage within the same column bearing different superscripts are significantly different (*p* < 0.05).

**Table 3:** Variation of pH in culture medium.

During Growth Phase	pH of Medium with			
	NaNO_3_	NaNO_2_	NH_4_Cl	CH_4_N_2_O
Day 0 (culture medium without cells)	8.50 ± 0.23 ^a^	8.69 ± 0.19 ^a^	7.87 ± 0.21 ^a^	8.67 ± 0.30 ^a^
Day 16 (culture medium before harvesting)	9.02 ± 0.25 ^a^	8.95 ± 0.21 ^a^	4.2 ± 0.14 ^b^	3.7 ± 0.13 ^b^

^ab^ Values for each stage within the same column bearing different superscripts are significantly different (*p* < 0.05).

**Table 4:** Changes in biomass productivity of *N. shiloi* and variation in the fucoxanthin content under oxidative stress conditions.

Oxidative Stress Sources in Seawaterbg-11	Fucoxanthin (mg/gDW)	Specific Growth Rate (day^−1^)	Dry Weight (g/L)
no^.^OH and ^1^O_2_	50.17 ± 1.02 ^d^	0.166 ± 0.03 ^a^	0.66 ± 0.01 ^a^
0.1 mM H_2_O_2_ + 0.1 mM Fe^2+^	58.20 ± 1.16 ^c^	0.142 ± 0.01 ^c^	0.15 ± 0.03 ^d^
0.1 mM NaClO + 0.1 mM Fe^2+^	65.14 ± 1.95 ^b^	0.152 ± 0.00 ^b^	0.28 ± 0.01 ^c^
0.1 mM H_2_O_2_ + 0.1 mM NaOCl	97.45 ± 2.64 ^a^	0.163 ± 0.04 ^a^	0.48 ± 0.02 ^b^

^abcd^ Values for each stage within the same column bearing different superscripts are significantly different (*p* < 0.05).

## Data Availability

All data generated or analyzed during this study are included in the article.

## Sample Availability

Samples of the brown microalge are available from the authors.
